# Diagnostic Value of Tears

**Published:** 1848

**Authors:** M. Trousseau


### Article VI.—*On the Diagnostic Value of Tears in Infantile Diseases.* By M. Trousseau.

The author states as a general rule, that when the infant sheds tears it is not dangerously ill; and, on the contrary, the absence of weeping indicates a severe disease. He regards this to be so true as to deserve to be considered as an aphorism. He does not deny, however, that there may be exceptions.—*Gazette des Hopitaux*, and *Revue Med.-Chirurg.*, Jan. 1848.

